# Genetic Mechanisms and Adaptive Benefits of Anthocyanin Red Stigmas in a Wind-Pollinated Tree

**DOI:** 10.1093/molbev/msaf040

**Published:** 2025-02-10

**Authors:** Wei-Hao Wang, Susanne S Renner, Hao-Sheng Liu, Liu-Feng Dai, Cai-Jin Chen, Yi Zhang, Bo-Wen Zhang, Da-Yong Zhang, Wei-Ning Bai

**Affiliations:** Ministry of Education Key Laboratory for Biodiversity Science and Ecological Engineering, College of Life Sciences, Beijing Normal University, Beijing 100875, China; Department of Biology, Washington University, Saint Louis, MO 63130, USA; Ministry of Education Key Laboratory for Biodiversity Science and Ecological Engineering, College of Life Sciences, Beijing Normal University, Beijing 100875, China; Key Laboratory of Cell Proliferation and Regulation Biology of Ministry of Education, College of Life Sciences, Beijing Normal University, Beijing 100875, China; Center for Biological Science and Technology, Zhuhai-Macao Biotechnology Joint Laboratory, Advanced Institute of Natural Science, Beijing Normal University, Zhuhai 519087, China; Ministry of Education Key Laboratory for Biodiversity Science and Ecological Engineering, College of Life Sciences, Beijing Normal University, Beijing 100875, China; Key Laboratory of Cell Proliferation and Regulation Biology of Ministry of Education, College of Life Sciences, Beijing Normal University, Beijing 100875, China; Ministry of Education Key Laboratory for Biodiversity Science and Ecological Engineering, College of Life Sciences, Beijing Normal University, Beijing 100875, China; Ministry of Education Key Laboratory for Biodiversity Science and Ecological Engineering, College of Life Sciences, Beijing Normal University, Beijing 100875, China; Ministry of Education Key Laboratory for Biodiversity Science and Ecological Engineering, College of Life Sciences, Beijing Normal University, Beijing 100875, China

**Keywords:** anthocyanin, photoprotection, red stigmas, transposon insertions, ubiquitin ligase gene MIEL1

## Abstract

Anthocyanin accumulation in leaves or flowers mitigates photooxidation damage from reactive oxygen species (ROS) and functions in plant/animal interactions. Among the most conspicuously anthocyanin-accumulating tissues are stigmas, especially in wind-pollinated trees. In the walnut genus (*Juglans*), yellow stigmas are ancestral, but a few species have dark red stigmas. We have used a natural *F*_1_ hybrid resulting from crosses between yellow stigma and red stigma species to investigate the genetic basis of the red stigmas. We found that a *Copia* transposable element (TE) insertion in the ubiquitin-protein ligase gene *MIEL1* suppresses its expression in stigmas through RNA-directed DNA methylation and has gone to fixation in red stigma species. A younger *Gypsy* TE insertion fully inhibits *MIEL1* expression, but is not fixed, explaining the color segregation in hybrid populations. Based on reference genomes and whole-genome sequencing data representing 20 of the 22 species of *Juglans*, we traced the evolution of *MIEL1*, finding the insertions in all consistently red stigma species. Red stigmas had lower levels of ROS than yellow stigmas, and population genetic data reveal strong positive selection on the TE-bearing *MIEL1* allele. In combination, these results suggest that anthocyanin-accumulating stigma tissues support pollen germination and growth by protecting cells from ROS.

## Introduction

Anthocyanins are produced in many reproductive and vegetative tissues and function in the mitigation of oxidative stress from bright sunlight, damage from herbivorous insects, and as color signals in plant/animal interactions (Gould et al. 1995, 2018; Niu et al. 2017; Sapir et al. 2021). Studies of anthocyanin-accumulating floral tissues have mostly focused on the role of red or purple petal colors in pollinator signaling and warmth accumulation in bowl-shaped dark purple flowers (van der Kooi et al. 2019), with less attention paid to red stigmas in wind-pollinated species (but see Meng et al. 2021; Devos et al. 2023). Yet red stigmas are common in wind-pollinated trees and grasses, including species of *Acer*, *Alnus*, *Carpinus*, *Coriaria*, *Corylus*, and *Myrica*, *Ostryopsis*, *Pterocarya*, *Tripsacum*, and many other genera. Their dark red styles and stigmas might be adaptive by increasing radiant energy absorption, which helps pollen germination and pollen tube elongation, processes that are highly sensitive to cold (Zinn et al. 2010), but it is unlikely that warmth accumulation during sunny hours would persist during the night. In bee-pollinated *Acer pictum*, which has individuals with red or yellow stigmas, experiments have shown that the red stigma morph has better pollen germination and fruit set than the greenish-yellow morph, but stigma temperatures or other biochemical effects were not investigated (Yang et al. 2015). Anthocyanins are flavonoids, and experiments in tomato have shown that anthocyanin-reduced mutants have impaired pollen germination and tube growth, likely because flavonols scavenge reactive oxygen species (ROS) and thereby act as antioxidants (Muhlemann et al. 2018).

**Fig. 1.**
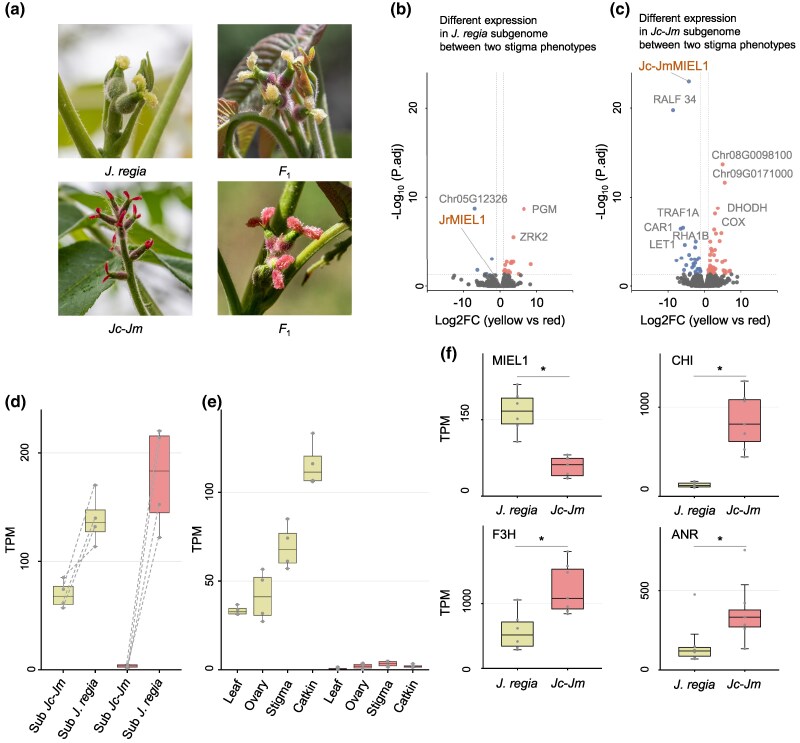
Phenotypic variation and differential gene expression in *Juglans* stigmas. a) Red and yellow stigmas of different *F*_1_ hybrid trees and their parents. b and c) Volcano plots of DEGs in the two subgenomes (see supplementary fig. S1, Supplementary Material online for experimental details), with red dots representing upregulated genes and blue dots downregulated genes. d) *MIEL1* gene expression levels (TPM) in yellow and red stigmas of *F*_1_ hybrids across the two subgenomes (“sub”). e) *Jc-Jm MIEL1* gene expression levels (TPM) in leaf, ovary, stigma, and catkin tissue of yellow stigma hybrid trees (yellow boxes) and red stigma hybrid trees (red boxes). f) Expression levels (TPM) of *MIEL1* and three genes (CHI, F3H, and ANR) in the anthocyanin pathway that are negatively correlated with *MIEL1* (supplementary table S2, Supplementary Material online). Statistical significance was determined using Wilcoxon rank-sum test (one-tailed), and star indicates *P* < 0.05.

The genus *Juglans*, with some 22 accepted species (Zhang et al. 2019: Fig. S1 shows all species with their geographic ranges), presents an opportunity to dissect the genomic mechanism of anthocyanin accumulation in stigmas because of a natural hybrid that is polymorphic for stigma color. All walnut flowers are unisexual, wind-pollinated, and lack petals. The large bilobed stigmas are yellow in most species, but red in four, namely *Juglans ailantifolia*, *Juglans cathayensis*, and *Juglans mandshurica* from eastern Asia and *Juglans cinerea* from eastern North America. Phylogenetic research has shown that the red stigma species form a clade (Zhang et al. 2019). The naturally occurring *F*_1_ hybrid, which has the species name *Juglans hopeiensis*, results from recurrent crosses between the yellow stigma species Persian walnut (*Juglans regia*) and hybrid individuals of the red stigma species *J. cathayensis* and *J. mandshurica* (Zhang et al. 2021). In earlier research, we extensively sampled the broad hybrid zone of *J. cathayensis* and *J. mandshurica* in the Beijing and Hebei region (Xu et al. 2021). This zone comprises cooccurring and coflowering trees with either red or yellow stigmas (Fig. 1a), a segregation that affords an opportunity to investigate the genetic basis of the color polymorphism and to test whether red stigmas have lower levels of ROS than do yellow stigmas. Mature trees of the parental species, as well as the hybrid, can be distinguished by a combination of leaf and fruit characters (Zhang et al. 2021).

We therefore measured stigma ROS levels and temperatures during sun light and used genomic analyses, transcriptome sequencing of stigma, ovary, catkin, and leaf tissue, transient transformation experiments, and methylation analyses to investigate the pathway leading to the red stigmas. Based on genomic data from 20 of the 22 accepted species, we also studied the evolution of specific transposon insertions in introns of the *MIEL1* gene, revealed here as regulating anthocyanin accumulation. *MIEL1* belongs to a gene family in plants that facilitates protein degradation through physical interactions with a target substrate (Shu and Yang 2017; Traver and Bartel 2023).

## Results

### Sampling and Genome Survey of Parents and *F*_1_ Hybrids with Red or Yellow Stigmas

We previously showed that the nomenclatural species *J. hopeiensis* consists exclusively of first-generation (*F*_1_) hybrids between *J. regia*, which has consistently yellow stigmas, and *J. cathayensis*/*J. mandshurica* hybrids (hereafter called *Jc-Jm*), with consistently red stigmas (Zhang et al. 2021). In a natural population near Beijing, we identified four *F*_1_ hybrid individuals with yellow stigmas and four with red stigmas (Fig. 1a). Genomic surveys of the eight individuals revealed high levels of heterozygosity (5.43% to 5.90%), allowing us to separate the two subgenomes that coexist in every *F*_1_ hybrid, the *J. regia* subgenome and *Jc-Jm* subgenome. After polishing and scaffolding with third- and second-generation whole-genome sequencing data, we obtained eight *F*_1_ haplotype-resolved genome assemblies (four from red and four from yellow stigma individuals), with sizes ranging from 931 to 969 Mb. To obtain gene expression profiles, we also performed RNA sequencing at the flowering stage in April 2023 for fresh samples of stigma, ovary, catkin, and leaf from the same eight hybrid individuals and from parental *J. regia* and *Jc-Jm* individuals.

### Allele-Specific Expression Profiles Revealed the *MIEL1* Gene as Mediating Stigma Color

Approximately 39.52% to 40.10% of RNA sequencing reads could be unambiguously aligned with and thus assigned to the *J. regia* subgenome, while 39.33% to 41.04% could be assigned to the *Jc-Jm* subgenome (supplementary table S1, Supplementary Material online). We then conducted differential expression analyses, using four replicates each for the hybrids’ stigma color morphs and comparing expression in red stigma trees with that in yellow stigma trees as illustrated in supplementary fig. S1, Supplementary Material online. The red and yellow stigmas differed by 22 differentially expressed genes (DEGs) of the *J. regia* subgenome, of which 18 were upregulated and 4 were downregulated in the red stigmas (Fig. 1b), and by 71 DEGs of the *Jc-Jm* subgenome, of which 41 were upregulated and 30 downregulated (Fig. 1c). Among these DEGs, the RING E3 ubiquitin-protein ligase *MIEL1* stood out as significantly downregulated in the *Jc-Jm* subgenome (Fig. 1c). Notably, *MIEL1* did not show differential expression in the *J. regia* subgenome across stigma colors (Fig. 1b). Further comparison of *MIEL1* expression levels between subgenome alleles consistently revealed lower expression of *Jc-Jm MIEL1* compared to *JrMIEL1* (Fig. 1d).

We next compared the expression levels of orthologous genes using additional fresh stigma samples from the parental genomes, *J. regia* (yellow stigma parent) and *Jc-Jm* (red stigma parent). Among 21,728 orthologous genes identified between the parental genomes, a total of 4,534 genes showed significantly differential expression, with 2,298 expressed more highly in *Jc-Jm* and 1,894 more highly in *J. regia* (supplementary fig. S2, Supplementary Material online). Among these, *MIEL1* also showed significantly lower expression in *Jc-Jm*, with a log fold change (Logfc) of −1.63 and a false discovery rate (FDR) of 7.22 × 10^−9^, consistent with the expression pattern observed between subgenome alleles in the hybrid.

To assess the consistency of *MIEL1* expression across tissues in the hybrid, we examined four tissue types: stigma, leaf, ovary, and catkin. In individuals with red stigmas, *Jc-Jm MIEL1* expression across all tissues was low (Fig. 1e), suggesting a systemic regulatory influence on *MIEL1* gene expression rather than a localized effect.

### *MIEL1* Negatively Regulates Anthocyanin Accumulation

We calculated correlation coefficients between *MIEL1* and anthocyanin structural genes using transcriptome data from *J. regia* and *Jc-Jm*, with the analysis conducted separately for each species across four different tissues. This revealed that the expression of three anthocyanin structural genes (Anthocyanidin reductase [ANR], Chalcone-flavanone isomerase [CHI], and Flavanone 3-hydroxylase [F3H]) was significantly negatively correlated with *MIEL1* in both parents (supplementary table S2, Supplementary Material online), with expression levels significantly higher in the stigma of the red stigma parent *Jc-Jm* than the yellow stigma parent *J. regia* (Fig. 1f; supplementary table S3, Supplementary Material online).

### Transposon Insertions in the Introns of *MIEL1* Alleles in Red Stigma and Yellow Stigma Trees

**Fig. 2.**
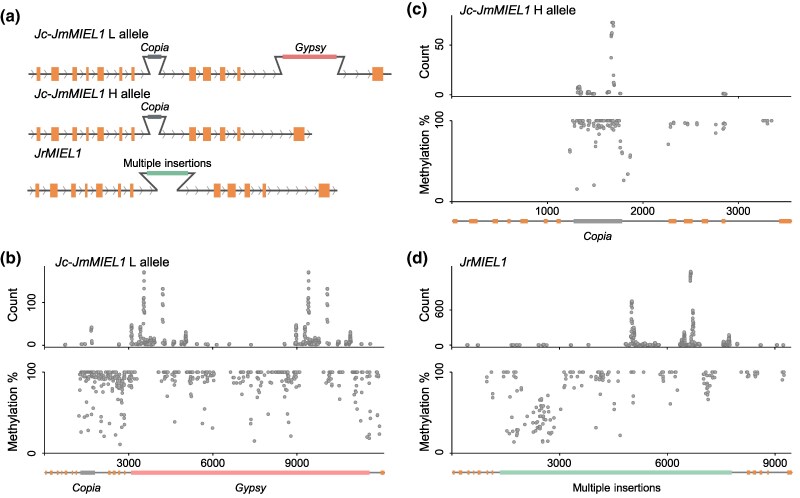
*MIEL1* structure, methylation levels, and small RNA target sites. a) Schematic representation of the *Gypsy* transposon-bearing *Jc-Jm MIEL1* L allele in a red stigma hybrid tree, the *Gypsy* transposon-free H allele in a yellow stigma hybrid tree, and the *JrMIEL1* allele in both morphs. Orange squares represent the CDS regions. b to d) Methylation levels and enrichment of small RNAs targeting the *Jc-Jm MIEL1* L allele, the *Jc-Jm MIEL1* H allele, and the *JrMIEL1* in red stigma *F*_1_ hybrids.

To confirm whether genetic variations at the DNA level were responsible for the differing expressions of the *Jc-Jm MIEL1* gene, we analyzed the full-length sequence of *Jc-Jm MIEL1* in the *F*_1_ hybrids. Using *Juglans nigra* as an outgroup, we identified two alleles of *Jc-Jm MIEL1* in the eight *Jc-Jm* subgenomes that differed by an over 8.5 kb insertion in the 11th intron (Fig. 2a). The allele from the four individuals with red stigma (*Jc-Jm MIEL1* L) had the 8.5 kb insertion, whereas the allele from the four individuals with yellow stigma (*Jc-Jm MIEL1* H) lacked this insertion (supplementary figs. S3 and S4, Supplementary Material online). The transposable element (TE) annotation for this insertion revealed a complete long terminal repeat (LTR)-type transposon classified within the Tekay subfamily of the *Ty3*-*Gypsy* superfamily, characterized by 2,639 base pair LTRs. To accurately identify the nucleotide sequences of both LTRs, primers were designed to amplify the two LTR sequences (Materials and Methods). It turned out they differed by only four nucleotides, indicating a relatively recent insertion event.

Additionally, another insertion of 517 bp was identified in the seventh intron of both alleles of *Jc-Jm MIEL1* (Fig. 2a), as well as in another published *J. mandshurica* genome (Li et al. 2022). This insertion was annotated as an incomplete *Copia* transposon. Comparison with homologous complete *Copia* sequences revealed 104 SNPs and 22 indels (<50 bp) (supplementary fig. S5, Supplementary Material online), suggesting that this insertion is older than the just-described *Gypsy* insertion.

In the *J. regia* subgenome of the hybrid and in published *J. regia* genomes (Marrano et al. 2020; Zhang et al. 2020; Han et al. 2024), an insertion of approximately 6 kb in length was identified within the seventh intron (Fig. 2a). This insertion includes seven types of transposons and some unclassified repeat regions. It starts with a 1,554 bp LINE and ends with an 808 bp unclassified repeat region. Different *J. regia* samples show small variations in this insertion (supplementary fig. S6, Supplementary Material online).

### Methylation Patterns and Small RNA Target Sites Support the Regulatory Role of the Transposon Insertions in *MIEL1*

Transposon insertions can increase gene methylation levels when they occur within or near genes, often mediated by small RNAs through RNA-directed DNA methylation (RdDM), and influence gene expression. Therefore, we examined both the methylation levels and the small RNA levels of *MIEL1*. First, we predicted potential CpG islands among the three alleles. In the *Jc-Jm MIEL1* H allele, one CpG island was identified within the 517 bp *Copia* insertion (supplementary fig. S7, Supplementary Material online). In the *Jc-Jm MIEL1* L allele, three CpG islands were identified, including the one also found in the *Jc-Jm MIEL1* H allele and two others with lengths of 512 bp on the *Gypsy* LTRs (supplementary fig. S8, Supplementary Material online). No CpG islands were identified in the *JrMIEL1* allele.

Next, we conducted whole-genome bisulfite sequencing of stigma tissue from three hybrid trees. We calculated cytosine methylation levels across different insertion regions, focusing on areas where the reads could be precisely mapped, as transposons often appear elsewhere in the genome, making it difficult to accurately determine the origin of some reads. The hybrid's *Jc-Jm MIEL1* L allele had high levels of methylation in its *Copia* and *Gypsy* insertions. Thus, 41.35% (98 sites, including 64 CG, 34 CHG, and 0 CHH sites) of the cytosines in its *Copia* insertion and 29.69% (312 sites, including 185 CG, 122 CHG, and 5 CHH sites) of the cytosines in its *Gypsy* insertions were methylated. In the H allele, 38.22% (91 sites, including 59 CG, 31 CHG, and 1 CHH sites) of the cytosines in the *Copia* insertion were methylated; there were no *Gypsy* insertions. The hybrid's *JrMIEL1* allele insertions had significantly lower methylation levels, with 10.23% (165 sites, including 42 CG, 120 CHG, and 3 CHH sites) of cytosines methylated in hybrid trees with red stigma and 11.47% (255 sites, including 85 CG, 160 CHG, and 10 CHH sites) in hybrid trees with yellow stigma. We also analyzed the cytosine methylation levels of CG, CHG, and CHH in the insertions of three different alleles. CG methylation levels were consistently high in the insertions of all three alleles, but the proportion of CG methylation was significantly higher in the *Jc-JmMIEL1* L and *Jc-JmMIEL1* H alleles compared to the *JrMIEL1* allele insertions (supplementary fig. S9, Supplementary Material online). CHG methylation levels were significantly higher in the insertions of the *Jc-JmMIEL1* L and *Jc-JmMIEL1* H alleles relative to the *JrMIEL1* allele insertions, and the proportion of CHG methylation was also higher in the *Jc-JmMIEL1* L and *Jc-JmMIEL1* H alleles than in the *JrMIEL1*. CHH methylation levels remained very low across the insertions of all three alleles (supplementary fig. S9, Supplementary Material online). In summary, the methylation levels of the *Jc-JmMIEL1* L and *Jc-JmMIEL1* H alleles were significantly higher than those of the *JrMIEL1* allele.

We also quantified the small RNA levels in the hybrid to investigate whether specific small RNAs may be targeting *MIEL1*. A significant small RNA peak was found clustered in the *Copia* insertion site of both *Jc-Jm MIEL1* alleles (Fig. 2b and c). Additionally, for the *Jc-Jm MIEL1* L allele, significant small RNA peaks were clustered in the LTR region of the *Gypsy* transposon intron (Fig. 2b). Similarly, *JrMIEL1* exhibited some small RNA enrichment regions (Fig. 2d); however, there was no difference between red and yellow stigmas.

Lastly, we quantified methylation and small RNA levels of the *MIEL1* gene in the parental species (*Jc-Jm* and *J. regia*). The *Jc-Jm MIEL1* H allele and the *JrMIEL1* allele in the parents displayed the same patterns as in the hybrid (supplementary fig. S9, Supplementary Material online). Thus, 40.25% (95 sites, including 61 CG, 32 CHG, and 2 CHH sites) of the cytosines in the *Copia* insertion of the *Jc-Jm MIEL1* H allele were methylated, while only 10.12% (234 sites, including 99 CG, 133 CHG, and 2 CHH sites) of the cytosines of the *JrMIEL1* allele insertions were methylated. The *Jc-Jm MIEL1* H allele in *Jc-Jm* stigma samples had one additional small RNA peak within the *Copia* insertion that was absent in the hybrid. The *J. regia* small RNA enrichment pattern was the same as in the hybrid.

### Evolution of *MIEL1* in *Juglans*

To investigate the evolution of *MIEL1*, we relied on 11 *Juglans* reference genomes, 7 representing the yellow stigma clade (Marrano et al. 2020: *J. regia*; Ning et al. 2020: *Juglans sigillata*; Zhang et al. 2020: *J. regia*; Han et al. 2024: *J. regia*, *Juglans hindsii*, *J. nigra*, and *Juglans microcarpa*), 3 representing the red stigma clade (Stevens et al. 2018: *J. cathayensis*; Li et al. 2022: *J. mandshurica*; Guzman-Torres et al. 2023: *J. cinerea*), and 1 with variable stigma color, namely *Juglans californica* (Fitz-Gibbon et al. 2023). To these genomes, we added 90 whole-genome sequencing data sets for *J. ailantifolia*, *J. cathayensis*, and *J. mandshurica* (the red stigma clade) and 12 for *Juglans jamaicensis*, *Juglans major*, *Juglans mollis*, *Juglans neotropica*, *Juglans olanchana*, *Juglans pyriformis*, *Juglans mexicana*, *Juglans steyermarkii*, and *Juglans venezuelensis* (the yellow stigma clade). Together, these 20 species represent most of the currently accepted c. 22 species of *Juglans* (Zhang et al. 2019). We used *Cyclocarya paliurus* (Batal.) Iljinsk. (originally described as *Pterocarya paliurus* Batal.) as an outgroup based on the phylogeny obtained by Ding et al. (2023). *Cyclocarya paliurus* has yellow stigmas and lacks insertions in the *MIEL1* gene.

**Fig. 3.**
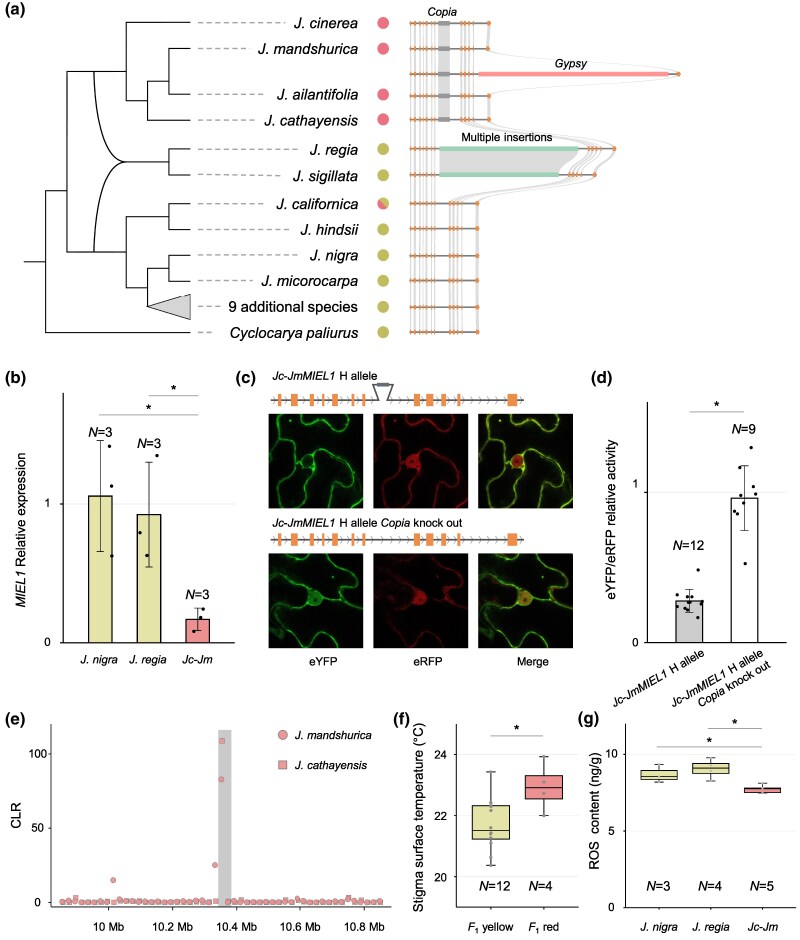
Evolution of the *MIEL1* gene in *Juglans*, RT-qPCR analysis, and test for selection. a) Evolution of *MIEL1* in 19 of the 22 species, excluding *J. hopeiensis*, which consists exclusively of *F*_1_ hybrids (Zhang et al. 2021), with red or yellow dots representing stigma colors. *Juglans californica* has red or yellow stigmas, but lacks insertions. The “9 additional species” were *J. jamaicensis*, *J. major*, *J. mollis*, *J. neotropica*, *J. olanchana*, *J. pyriformis*, *J. mexicana*, *J. steyermarkii*, and *J. venezuelensis*. b) RT-qPCR results of *MIEL1* in *J. regia*, *J. nigra*, and *Jc-Jm* stigmas. c) *Agrobacterium*-mediated transient transformation assay with tobacco leaves and the *Jc-Jm MIEL1* H allele or instead the *Jc-Jm MIEL1* H allele *Copia* knockout. d) eYFP/rYFP ratio analysis of different vectors. e) CLR test for selection across chromosome 10 in *J. mandshurica* and *J. cathayensis*, based on 1,500 grids and calculated using *SweeD*. The shaded gray areas indicate the region surrounding the *MIEL1* gene. f) Surface temperatures of yellow and red stigmas of hybrid trees measured at noon on 2024 April 15, when air temperatures were 19 to 20 °C. g) ROS concentration (ng/g) in red stigmas of *Jc-Jm* flowers and in yellow stigmas of *J. regia* and *J. nigra* flowers. Statistical significance was determined using Wilcoxon rank-sum test, and star indicates *P* < 0.05.

Results showed that the *MIEL1* gene of all sampled red stigma species displays a 517 bp *Copia* insertion in the seventh intron (Fig. 3a; supplementary figs. S10 and S11, Supplementary Material online), while among the yellow stigma species, only *J. regia* and *J. sigillata* exhibit multiple transposon insertion of approximately 6 kb in the seventh intron of the *MIEL1* gene (Fig. 3a; supplementary figs. S12, S13, and S14, Supplementary Material online). The American species *J. californica*, which has yellow, pink, or red stigmas, lacks any *Copia* or *Gypsy* insertion in its *MIEL1* gene, while the American species *J. cinerea*, which has consistently red stigmas, has it.

### *MIEL1* Expression Levels and Selection Analyses

To further validate our transcriptome results and explore the expression of the *MIEL1* gene in the two stigma color morphs, we conducted RT-qPCR experiments on red stigma hybrids (*J. cathayensis*/*J. mandshurica*) and two yellow stigma species, *J. nigra* and *J. regia*. This revealed significantly lower *MIEL1* expression levels in *Jc-Jm* compared to *J. nigra* and *J. regia* (Fig. 3b), matching the results of our transcriptome differential expression analysis. Interestingly, there was no significant difference in *MIEL1* expression between *J. regia* and *J. nigra*, despite the presence of TE insertions in the seventh intron of *JrMIEL1*. To control for dosage effects of the intron's presence, we constructed expression vectors driven by the 35S promoter for both the native *Jc-Jm MIEL1* H allele and a *Copia* knockout variant. Transient expression assays in *Nicotiana benthamiana* revealed that the plasmid lacking the *Copia* insertion had markedly higher expression levels than the plasmid containing the *Copia* insertion (Fig. 3c and d). This provides compelling evidence that the insertion of the *Copia* transposon is responsible for the observed decrease in gene expression.

Lastly, given that *Copia* elements are stably present in the red stigma species, likely due to positive selection, we conducted composite likelihood ratio (CLR) and Tajima's *D* (Tajima 1989) tests on the chromosome containing the *MIEL1* gene using the 40 whole-genome sequencing data sets of *J. mandshurica* and *J. cathayensis* (both with consistently red stigmas). Tajima's *D* analysis revealed a reduction to below −2 on chromosome 10 near the *Jc-JmMIEL1* gene (supplementary fig. S15, Supplementary Material online), which, combined with the CLR test detecting values within the top 1% in the same region (Fig. 3e; supplementary fig. S16, Supplementary Material online), provides strong evidence that MIEL1 has undergone positive selection.

To assess the presence and frequency of the *Jc-JmMIEL1* L allele in the parental species, we designed primers and amplified the relevant region in 48 *Jc-Jm* individuals. This revealed that 3 of the 48 individuals were heterozygous for this allele (supplementary figs. S17 and S18, Supplementary Material online), yielding an allele frequency of only 0.03125, suggesting that the newly formed allele, resulting from transposon insertions, is young and has not yet dispersed widely within the population.

### Daytime Temperatures and ROS Contents of Red and Yellow Stigmas

Around noon of 2024 April 15, we measured the temperatures of red and yellow stigmas on six *F*_1_ hybrid trees. The temperature of the red stigmas at 12 PM was 22.94 °C ± 0.7, while that of the yellow stigmas was 21.71 °C ± 0.83, with surrounding air temperatures of 19 to 20 °C (Wilcoxon rank-sum test, *P* = 0.0418; Fig. 3f). The concentration of ROS in the red stigmas of *Jc-Jm* flowers and the yellow stigmas of *J. regia* and *J. nigra* was quantified using three to five biological replicates per species. ROS levels in red stigma tissues of *Jc-Jm* were significantly lower than those in the yellow stigmas of *J. nigra* (Wilcoxon rank-sum test, *P* = 0.0357) and *J. regia* (Wilcoxon rank-sum test, *P* = 0.0159; Fig. 3g).

## Discussion

In this study, we investigate the molecular mechanisms underlying the yellow/red stigma color polymorphism observed in natural walnut hybrid trees near Beijing and Hebei, and the evolution of red stigmas in the entire *Juglans* genus. The results suggest an adaptive explanation for red stigmas in spring-flowering trees, based on photoprotection. We discovered that transposon insertions in the introns of the ubiquitin-protein ligase gene *MIEL1*, which negatively regulates anthocyanin synthesis by degrading transcription factors that control the expression of genes in the anthocyanin pathway (An et al. 2019; An et al. 2020; Liu et al. 2024), decrease its expression, which then affects the expression of downstream genes (ANR, CHI, F3H) in the anthocyanin pathway (Fig. 1f; supplementary table S2, Supplementary Material online).

Specifically, a methylated *Copia* transposon insertion in *MIEL1* reduces its expression as demonstrated by our *Agrobacterium*-mediated transient transformation experiments. The presence of small RNA within the inserted transposons suggests that the associated methylation may have been established through RdDM pathways. The *Copia* TE insertion has gone to fixation in red stigma species, while a younger *Gypsy* insertion that fully inhibits *MIEL1* expression is under positive selection, but not fixed, explaining the color segregation in hybrid populations: Hybrid individuals that inherit the *MIEL1* allele with both TEs have red stigmas, while individuals that inherit the allele with only the *Copia* insertion have yellow stigmas.

While the downregulation and eventual complete functional suppression of *MIEL1* thus emerges as controlling the anthocyanin accumulation, other genes in the anthocyanin pathway may also contribute to the red stigma phenotype in walnuts. For instance, anthocyanin 5-*O*-glucosyltransferase (*A5GT*) and anthocyanin 5-glucoside malonyl transferase (*5MAT*) show significant differential expression in *Jc-Jm* and *J. regia*, enhancing anthocyanin stability through glycosylation and acylation (supplementary table S3, Supplementary Material online). This warrants verification through the development of a transformation system in future research.

Since we only resequenced eight *F*_1_ hybrid genomes, our approach primarily focused on allele-specific expression to identify candidate genes. Variations in untranslated regions (UTRs) that affect mRNA stability, translation efficiency, or other posttranscriptional processes, as well as structural variations related to splicing, could also contribute to phenotypic variation (Wang et al. 2022; Kan and Li 2023). Future studies could integrate full-length transcriptomics or pangenome analysis with larger sample sizes to comprehensively examine both coding and noncoding regions.

We detected positive selection on the *MIEL1* allele in both Asian red stigma species, *J. mandshurica* and *J. cathayensis* (Fig. 3e); the American red stigma species *J. cinerea* was included in the genomic analyses (Fig. 3a), but not in the population-genetic analyses. The dark stigmas of these walnuts as well as many other wind-pollinated trees and grasses, such as species of *Acer*, *Alnus*, *Carpinus*, *Coriaria*, *Corylus*, *Myrica*, *Ostryopsis*, *Pterocarya*, and *Tripsacum*, may be adaptive in two ways: firstly, by reducing ROS levels in styles and stigmas that accumulate anthocyanin (Fig. 3g) and thus providing photoprotection. That anthocyanin accumulation supports pollen germination and growth by scavenging ROS and acting as an antioxidant has been shown in tomato (Muhlemann et al. 2018), and this may be especially important in sun-exposed thin-walled cells such as stigma papillae, style transmission cells, and pollen tubes, especially in wind-pollinated species that tend to have relatively large and exposed stigmas. Secondly, red stigmas and styles also absorb more radiant energy and during sunny days are therefore slightly warmer than yellow-greenish stigmas (Fig. 3f). While this may be beneficial for pollen germination and tube elongation, processes that are highly sensitive to cold (reviewed in Zinn et al. 2010), these tissues are too small to retain any warmth during the night.

## Materials and Methods

### Plant Sequencing and Genome Assemblies

Plant materials were sampled from a natural population located near Beijing, China; herbarium vouchers are listed in Zhang et al. (2019: Table S1). Genomic analysis focused on eight hybrid individuals (“*J. hopeiensis*”), with four replicates for each stigma phenotype. Fresh leaf tissues were used for DNA extraction, employing the CTAB method. The extracted DNA underwent sequencing using Oxford Nanopore Technologies (ONT) and Illumina platforms, each achieving a sequencing depth of over 30×. For Illumina sequencing, libraries were prepared using the VAHTS Universal Plus DNA Library Prep Kit for Illumina ND617. Sequencing was conducted on the Illumina NovaSeq 6000 platform in paired-end 150-base mode. For ONT sequencing, libraries were prepared using the Ligation Sequencing Kit 1D (PM) to generate an 8K-length library. Sequencing was performed on the PromethION48 platform using PromethION Flow Cells (FLO-PRO002) chips.

Genome heterozygosity was evaluated in a whole-genome survey using GenomeScope 2.0 (Ranallo-Benavidez et al. 2020) with Illumina reads. Contig-level assemblies were generated by integrating both short-read (Illumina) and long-read (ONT) data. Initially, genomes from the eight hybrid individuals were de novo assembled in haplotype mode using Flye (Kolmogorov et al. 2019) with ONT sequencing data. Subsequently, the assembled genomes underwent three rounds of correction using Pilon v1.24 (Walker et al. 2014), leveraging Illumina reads. To organize the assemblies into 32 pseudo-chromosomes for each hybrid sample, RagTag v2.1.0 (Alonge et al. 2022) was employed for scaffolding contigs based on the reference genomes of *J. regia* (Zhang et al. 2020) and *J. mandshurica* (Li et al. 2022).

To annotate TEs, we utilized EDTA v2.1.3 (Ou et al. 2019) on both a reference genome (Zhang et al. 2020; Li et al. 2022) and assembled genomes. The parameters used were ‘--species others --sensitive 1 --anno 1 --threads 5 --evaluate 1'. This approach yielded nonredundant transposon libraries and complete LTR retrotransposons for each subgenome. For complete LTR-type transposons that remained unclassified, TEsorter (Zhang et al. 2022) was employed to determine their LTR-type classification.

### RNA-seq Data Processing

Transcriptome sequencing was conducted across stigma, ovary, catkin, and leaf tissue from the same hybrid trees utilized for genomic sequencing. Additionally, stigma, ovary, catkin, and leaf tissue from the parent species, *J. regia* (6 replicates) and *Jc-Jm* (7 replicates), were included, resulting in a total of 84 samples. The RNA-Seq data were pooled and sequenced on the Illumina Novaseq6000 platform.

Transcriptome data from *J. regia* and *Jc-Jm* were initially aligned to their respective reference genomes. Genomic variant information was then extracted using samtools v1.19 (Danecek et al. 2021), and bcftools v1.1.7 (Danecek et al. 2021) was used to generate reference genome consensus sequences containing degenerate bases. Subsequently, BBSplit (BBMap v38.47; B. Bushnell, https://sourceforge.net/projects/bbmap/) was used to segregate reads from hybrid transcriptomes based on *J. regia* and *Jc-Jm* consensus genome sequences, retaining only perfectly split reads for allele-specific expression analysis. The split reads were mapped to their respective reference subgenomes using HISAT2 v2.2.1 (Kim et al. 2015). Gene expression levels were obtained using featureCounts v2.0.6 (Liao et al. 2014), followed by the computation of transcript per million (TPM) values. To mitigate the impact of highly DEGs, the TMM normalization method was applied by edgeR v4.2.0 (Robinson et al. 2010) to ensure appropriate adjustments.

For differential expression analysis of the parental species, we first utilized JCVI (Tang et al. 2024) to identify orthologous genes between *J. regia* and *J. mandshurica*. Subsequently, orthologous genes were identified through collinearity analysis using the same approach as used for the *F*_1_ individuals to analyze differential expression between the *J. regia* and *Jc-Jm*.

### Identification and Correlation Analysis of Anthocyanin Structural Genes

The identification of anthocyanin structural genes was conducted using KIPEs3 v0.3.5 (Rempel et al. 2023), focusing on the highest-scoring genes within the anthocyanin biosynthetic pathway. To investigate the regulatory mechanisms involving *MIEL1* in anthocyanin biosynthesis, we calculated Spearman’s and Pearson’s correlation coefficients between *MIEL1* and anthocyanin structural genes it appears to regulate (An et al. 2017), using TPM values from transcriptome data of stigma, ovary, catkin, and leaf tissues in *J. regia* and *Jc-Jm*. Significance testing was performed using a one-tailed test, and the Benjamini–Hochberg method was applied to control for FDR.

### Presence and Frequency of *Gypsy* Insertions

To validate the presence and frequency of *Gypsy* insertions in the parental population, the forward and reverse primers CDS11-F/GPS-CDS11-R were used. One primer targets the coding sequence (CDS) region of the *MIEL1* gene, while the other targets transposon insertion sites. Successful amplification of the target fragment confirms the presence of a *Gypsy* insertion. Since the studied species is diploid, additional primers CDS11-F/MIEL1-End-R were used to amplify the 11th intron. For individuals harboring the *Gypsy* insertion, failure to amplify the 11th intron indicates homozygosity for the insertion (both alleles carry the insertion). Conversely, successful amplification of the 11th intron suggests that the individual is heterozygous for the insertion. Moreover, primers Intron7-F/MIEL1-End-R were used to validate the *Gypsy* insertion in red stigma hybrid individuals by PCR. Our approach regarding *Copia* insertions in the *MIEL1* gene throughout the genus *Juglans* is described below.

### Methylation Prediction and Identification

Methylation and small RNA data were analyzed using three replicates each for the red and yellow stigmas of the hybrid, along with stigma tissue samples from the parental species. DNA extraction was performed using the TIANGEN Plant Genome DNA Kit, followed by fragmentation of the extracted DNA to sizes ranging from 200 to 400 base pairs using the Covaris S220. Subsequently, bisulfite treatment was applied to convert unmethylated cytosines to uracils, while methylated cytosines remained unchanged. After bisulfite treatment, DNA fragments were used to construct libraries with the Accel-NGS Methyl-Seq DNA Library Kit (Swift, USA, Catalog #:30096). Paired-end sequencing of the samples was conducted on the Illumina NovaSeq 6000 platform. Sequencing depth exceeded 60× for the hybrid and 30× for the parent species.

Firstly, CpG islands across the entire length of the *MIEL1* gene in the parents and the hybrid were predicted using Emboss CpGplot (Madeira et al. 2024). Predicted islands were defined by criteria including a length greater than 200 bp, a GC content exceeding 50%, and an observed/expected ratio above 0.60. To validate the presence of methylated sites within the predicted CpG islands, we used whole-genome bisulfite sequencing.

To analyze our methylation data, we used Bismark v0.24.2 (Krueger and Andrews 2011), which processes the reference genomes through conversion, modifying the genomes such that C is replaced with T and G with A. Subsequently, sequencing reads were aligned to these converted reference genomes using Bowtie2 v2.5.1 (Langmead and Salzberg 2012), followed by the removal of PCR duplicates. Finally, the methylation levels for all cytosine contexts (CG, CHG, and CHH) were extracted at each site. For *J. regia* and *Jc-Jm*, reads were mapped to the conversion genome of each species to obtain the methylation levels of each site. Additionally, to assess methylation levels within TE insertions in a red stigma hybrid, representative genomes derived from de novo assembly of red and yellow stigma samples were chosen as references.

### Small RNA Identification

For small RNA sequencing, total RNA served as the starting material for library preparation. First, 3′ and 5′ adapters were ligated to the respective ends of small RNAs, resulting in first-strand cDNA synthesis through hybridization with reverse transcription primers. The resulting double-stranded cDNA library was used for PCR amplification, and after purification and size selection, the library containing inserts ranged from 18 to 40 bp, suitable for Illumina sequencing using SE50 sequencing mode. Each sample was sequenced to yield a minimum of 10 million reads.

Raw sequencing reads underwent preprocessing using fastp v0.23.4 (Chen 2023) to perform adapter trimming and quality control. Trimmed reads were subsequently aligned to the reference sequence using Bowtie (Langmead et al. 2009), employing different parameters tailored for specific analyses. For individuals lacking intact LTR insertions, parameters ‘-v 1 -m 1 -k 1 --best –strata’ were used. Conversely, for individuals with intact LTR insertions, parameters ‘-v 0 -a --best –strata’ were applied. These distinct mapping strategies were employed to identify potential sites targeted by small RNAs. The resulting alignments were then analyzed using samtools v1.19 (Danecek et al. 2021) to assess coverage depth at various positions along the sequence.

### Vector Construction and *Agrobacterium*-Mediated Transient Transformation of Tobacco

To generate gene expression constructs for the *Jc-Jm MIEL1* H allele (which contained the *Copia* insertion), PCR amplification was used to sequence its full length, using the primers *Jc-Jm*-eYFP-F/*Jc-Jm*-eYFP-R, as well as sequences flanking the transposon insertion site with the primers Copia-F/Copia-R. The gene fragments were cloned into the pFGC vector containing eYFP under the 35S promoter between restriction sites of XhoI via infusion cloning (Vazyme) to generate *p35S: Jm-JmMIEL1-GFP*, *p35S: Jm-JmMIEL1 knock out-GFP*. The reference plasmid, GIP1, contained the red fluorescent protein gene. All the binary vectors described above were transformed into *Agrobacterium* strain GV3101. Then GV3101 was cultured in liquid Luria–Bertani medium overnight. At OD_600_ = 1.2, the bacteria were collected by centrifugation at 4,000 × *g* for 5 min and resuspended in deionized water to a final concentration of OD_600_ = 1.0. The resuspended bacteria were mixed in equal volumes and injected into young leaves of *N. benthamiana* using 1 mL syringes without needles. The protein expression of the infiltrated leaves was checked 2 d later through confocal microscope. Images were acquired using Zeiss 880 laser scanning confocal microscope equipped with 63 × 1.4NA objective.

### Identification of *Copia* Insertions Throughout the Genus *Juglans*

To investigate the evolution of the *MIEL1* gene in the genus *Juglans*, the gene sequence of *Jc-Jm MIEL1* was used as a query in blastn (Altschul et al. 1990) searches focusing on the 11 published *Juglans* reference genomes as cited in our Results section and newly generated WGS data for another 9 species, viz. *J. jamaicensis* (2 samples); *J. major* (1); *J. mollis* (1); *J. neotropica* (2); *J. olanchana* (2); *J. pyriformis* (1); *J. mexicana* (1); *J. steyermarkii* (1); and *J. venezuelensis* (1). Together, these 20 species represent most of the currently accepted species of *Juglans*. For the new whole-genome sequencing data, we relied on the HybPiper v2.1.6 (Johnson et al. 2016) assembly pipeline to obtain the full-length *MIEL1* gene with the complete gene sequence of *J. nigra* as the target. The process involved several steps: initially querying and sorting reads based on the target gene, followed by assembling these reads into contigs using SPAdes (Prjibelski et al. 2020), and subsequently aligning these contigs to a reference genome. Potential insertion fragments were then scrutinized by extracting the supercontigs to detect any insertions in the *MIEL1* gene.

### RNA Extraction and RT-qPCR

To investigate the impact of TEs on the expression of the *MIEL1* gene in *Juglans*, we sampled *Jc-Jm* (with red stigmas), and *J. regia* and *J. nigra* (both with yellow stigmas) and conducted RT-qPCR analysis with three stigma tissue samples from each species. Both *Jc-Jm* and *J. regia* were again sampled from the same population near Beijing, while *J. nigra* was obtained from Institute of Botany of the Chinese Academy of Sciences, Beijing, China. Total RNA was extracted using TRIzol. For cDNA synthesis, 1 µg of total RNA was used, and the resultant cDNA served as the template for subsequent RT-qPCR reactions. The RT-qPCR assays utilized TAKARA TB Green Premix Ex Taq II. Internal control genes included *J. regia* Actin-7 (LOC109008915) and orthologous genes in *J. nigra* and *Jc-Jm*. Data analysis followed the 2^−ΔΔCt^ method (Livak and Schmittgen 2001), normalizing expression levels to the internal controls. Primers used in this part of our study are listed in supplementary table S4, Supplementary Material online.

### Positive Selection Detection on the *MIEL1* Allele of the Red Stigma Species

To assess potential selection on the *MIEL1* gene of the red stigma species *J. mandshurica* and *J. cathayensis*, we relied on published population-level sequencing data (Xu et al. 2021). We trimmed the data to eliminate low-quality reads and then aligned the remaining high-quality reads to the *J. mandshurica* genome using the BWA-MEM algorithm of BWA v0.7.18 (Li and Durbin 2009). Variants were called considering sites with sequencing depths exceeding 6 and being less than 100 using bcftools v1.21 (Danecek et al. 2021). Positive selection analysis was performed on chromosome 10, where the *MIEL1* gene is located, by calculating Tajima's *D* (Tajima 1989) using 20-kb sliding windows with vcftools v0.1.16 (Danecek et al. 2011) and CLR with 1,500 grids using *SweeD* v3.1 (Pavlidis et al. 2013).

### Stigma Temperature Measurements in the Field

Temperature measurements were conducted at 12 PM on 2024 April 15 in a plantation of hybrid walnuts (“*J. hopeiensis*”) near Beijing, utilizing an infrared thermal imaging camera. The ambient air temperature during the measurements was 19 to 20 °C. We took the temperatures of 4 red stigmas from 2 trees and 12 yellow stigmas from 4 trees, taking 3 temperature readings per stigma. A Wilcoxon test was used to assess whether there was a significant difference in the average surface temperature between stigma colors.

### ROS Content Measurements in Stigmas

We measured the ROS content in red stigmas of *Jc-Jm* flowers and in yellow stigmas of *J. regia* and *J. nigra* utilizing three to five biological replicates per group. The samples were obtained from the same population as the RT-qPCR experiment. The ELISA method, using a double-antibody sandwich technique, quantifies ROS levels. Samples were incubated with HRP-labeled ROS antibodies. TMB substrate was added, resulting in a color change from blue to yellow under HRP catalysis. Absorbance (OD) was measured at 450 nm, and ROS concentrations were calculated from a standard curve. Each assay was performed in triplicate, with average values reported as the final ROS content. Wilcoxon tests were employed to determine significant differences in ROS levels among groups.

## Supplementary Material

msaf040_Supplementary_Data

## Data Availability

The DNA sequencing, RNA sequencing, small RNA sequencing, and whole-genome bisulfite sequencing data generated in this study are available on GenBank under accession number PRJNA356989 and at the National Genomics Data Center under BioProject number PRJCA010540 (https://ngdc.cncb.ac.cn/bioproject/browse/PRJCA010540). The assembled genomes are available at the National Genomics Data Center under BioProject number PRJCA010540 and can also be accessed at our website (http://cmb.bnu.edu.cn/juglans).
