# Supplementary material for: Genetic Mechanisms and Adaptive Benefits of Anthocyanin Red Stigmas in a Wind-Pollinated Tree
Source: Mol Biol Evol. 2025 Feb 10;42(3):msaf040. doi: 10.1093/molbev/msaf040 (PMC11879928; doi:10.1093/molbev/msaf040)
Supplement: msaf040_Supplementary_Data [file msaf040_supplementary_data.pdf]

Supporting Information for

Genetic mechanisms and adaptive benefits of anthocyanin red stigmas in a wind-pollinated tree

Wei-Hao Wang[1], Susanne S. Renner[2], Hao-Sheng Liu[1], Liu-Feng Dai[3, 4], Cai-Jin Chen[1], Yi Zhang[3], Bo-Wen Zhang[1*], Da-Yong Zhang[1*] & Wei-Ning Bai[1*]

[1]Ministry of Education Key Laboratory for Biodiversity Science and Ecological Engineering, College of Life Sciences, Beijing Normal University, 100875 Beijing, China.

[2]Department of Biology, Washington University in Saint Louis, MO 63130, USA

[3]Key Laboratory of Cell Proliferation and Regulation Biology of Ministry of Education, College of Life Science, Beijing Normal University, 100875 Beijing, China

[4]Center for Biological Science and Technology, Zhuhai-Macao Biotechnology Joint Laboratory, Advanced Institute of Natural Science, Beijing Normal University, Zhuhai 519087, China

*Corresponding authors: zhangbw@bnu.edu.cn; zhangdy@bnu.edu.cn; baiwn@bnu.edu.cn

This PDF file includes:

Figures S1 to S18
Tables S1 to S4

Table of contents

Figure S1 Differential expression analysis of stigma phenotypes in F_1 hybrids **1**
Figure S2 Volcano plot of differentially expressed genes in stigma of *J. regia* and *Jc-Jm* **1**
Figure S3 Multiple sequence alignment of the *Jc-JmMIEL1* H and L alleles in F_1 hybrids with different stigma color phenotypes..........**2**
Figure S4 Confirmation of *Gypsy* insertion in F_1 hybrids by PCR amplification **2**
Figure S5 Sequence alignment of the *Copia* insertion in *Jc-JmMIEL1* with homologous complete long-terminal-repeat retrotransposons..........**3**
Figure S6 Multiple sequence alignment of *JrMIEL1* based on the *J. regia* genomes and assembled F_1 hybrid genomes **3**
Figure S7 Identification of CpG islands in the *Jc-JmMIEL1* H allele **4**
Figure S8 Identification of CpG islands in the *Jc-JmMIEL1* L allele **5**
Figure S9 Proportion of cytosine context and its methylation levels in different allele insertions **6**
Figure S10 *Copia* insertions identified in *J. ailantifolia*, *J. mandshurica*, *J. cathayensis* and hybrid individuals from the hybrid zone of *J. mandshurica* and *J. cathayensis* (Xu et al. 2021) based on whole-genome sequencing data..........**7**
Figure S11 The *Copia* insertion in *J. cinerea*, *J. cathayensis*, and *J. mandshurica* based on their reference genomes as cited in the main text and using *Cyclocarya paliurus* as the outgroup **8**
Figure S12 Intron insertions in *J. regia* and *J. sigillata* based on their reference genomes as cited in the main text **8**
Figure S13 The *Copia* insertion in *J. jamaicensis*, *J. major*, *J. mollis*, *J. neotropica*, *J. olanchana*, *J. pyriformis*, *J. mexicana*, *J. steyermarkii*, and *J. venezuelensis* (all with yellow stigmas) based on whole-genome sequencing data as cited in the main text **8**
Figure S14 Absence of a *Copia* insertion in *J. hindsii*, *J. nigra*, *J. californica* (with variable stigma color), and *J. microcarpa* (both with yellow stigmas), using *J. mandshurica* (with red stigmas) as the reference **8**
Figure S15 Genome-wide Tajima`s D test results in 20-kb sliding windows across chromosome 10..........**9**
Figure S16 Genome-wide *SweeD* result with 1500 grid positions across chromosome 10 ....**10**
Figure S17 *Gypsy* insertion confirmation with PCR amplification in hybrid individuals of the red-stigma species *J. cathayensis* and *J. mandshurica* **11**
Figure S18 Identification of heterozygous or homozygous *Gypsy* insertions by PCR amplification **11**
Table S1 Assignment of the RNA-sequencing reads obtained from F_1 hybrids to its parents..........**12**
Table S2 Correlation coefficients between *MIEL1* and *Juglans* homologs of the anthocyanin-structural genes: Anthocyanidin Reductase (ANR), Chalcone-Flavanone Isomerase (CHI), and Flavonone 3-Hydroxylase (F3H)..........**13**
Table S3 Differentially expressed genes in the anthocyanin pathway across *J. regia* and the hybrid individuals of *J. mandshurica* and *J. cathayensis* **14**
Table S4 Primer sequences used in this study..........**15**

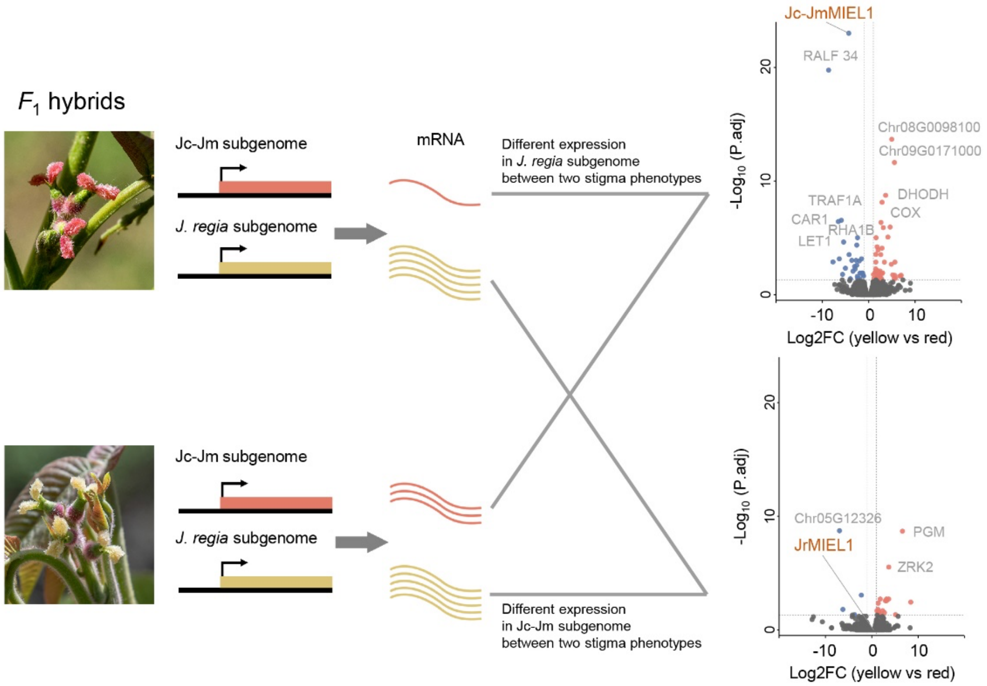

Fig. S1. Differential expression analysis of stigma phenotypes in F_1 hybrids.

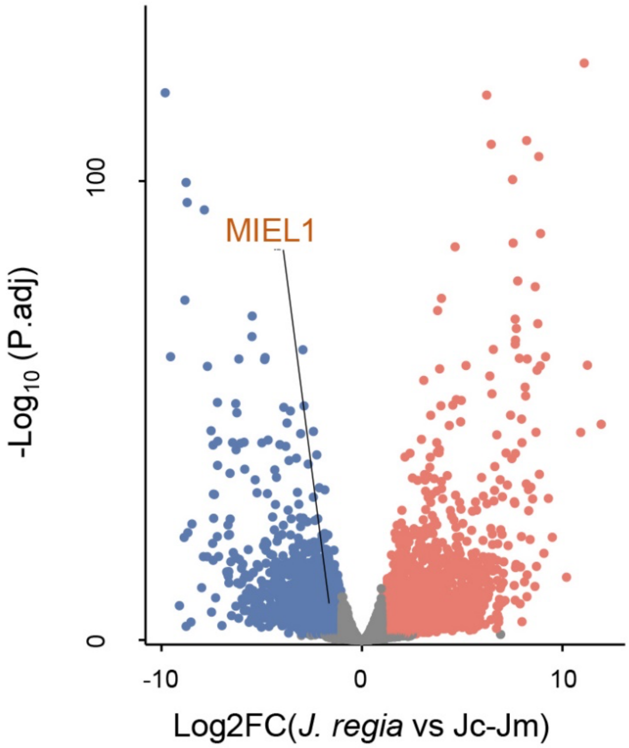

Fig. S2. Volcano plot of differentially expressed genes in stigmas of *J. regia* and *Jc-Jm*. Red (right) dots represent upregulated genes and blue (left) points represent downregulated genes. The *MIEL1* gene is significantly downregulated in *Jc-Jm* stigmas compared to *J. regia*.

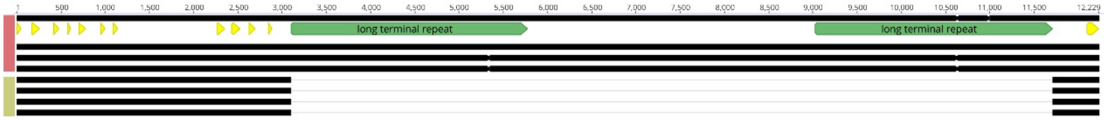

Fig. S3. Multiple sequence alignment of the *Jc-JmMIEL1* H and L alleles in F_1 hybrids with different stigma color phenotypes. Yellow arrows indicate coding sequence regions, and green arrows indicate long terminal repeats of *Gypsy* insertions. The top four sequences are from red stigma individuals, where *Gypsy* insertions are present in intron 11, and the bottom four from yellow stigma individuals, where *Gypsy* insertions are absent.

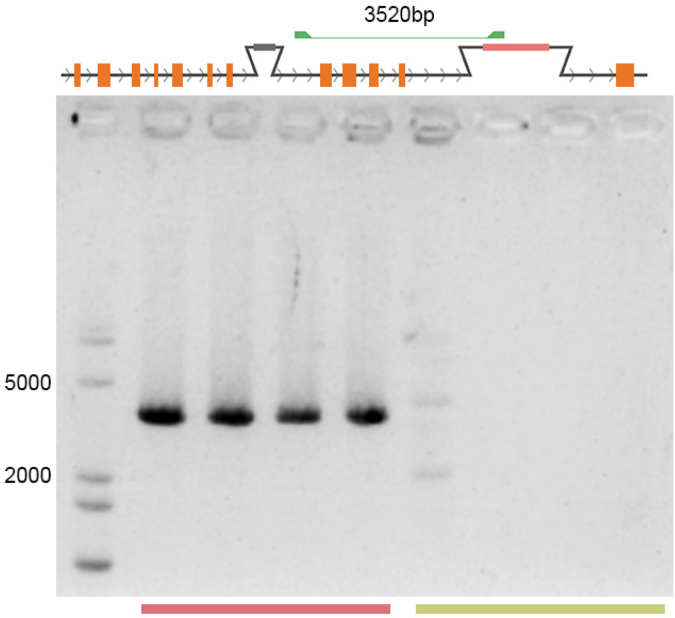

Fig. S4. Confirmation of *Gypsy* insertion in F_1 hybrids by PCR amplification. Primer locations are shown at the top, with orange rectangles indicating coding regions, pink indicating *Gypsy* insertions, and green indicating primer amplification regions. The left four lanes represent PCR amplification results from F_1 hybrids with red stigmas, while the right four lanes represent those with yellow stigmas. The results indicate the presence of a *Gypsy* insertion in intron 11 of the *Jc-JmMIEL1* gene in red-stigma F_1 hybrids, whereas this insertion is absent in yellow-stigma F_1 hybrids.

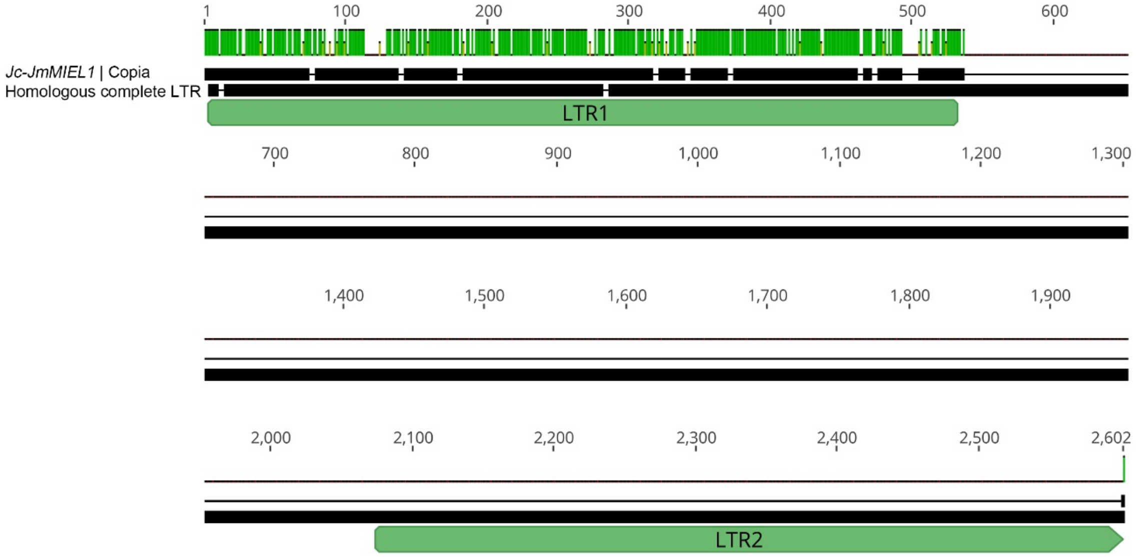

Fig. S5. Sequence alignment of the *Copia* insertion in *Jc-JmMIEL1* with homologous complete long-terminal-repeat retrotransposons. The alignment indicates that this segment of *Copia* in *Jc-JmMIEL1* corresponds to the long terminal repeat (LTR) region of its complete homolog. The comparison revealed 104 SNPs and 22 indels (<50 bp).

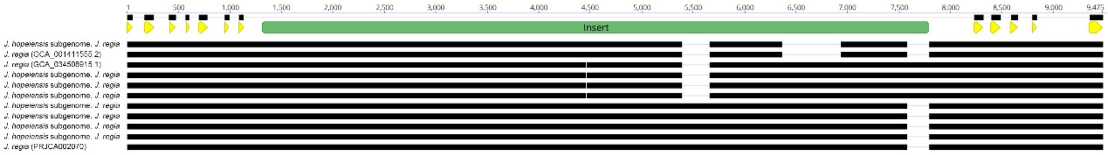

Fig. S6. Multiple sequence alignment of *JrMIEL1* based on the *J. regia* genomes and assembled F_1 hybrid genomes. Yellow arrows indicate coding sequence regions and green bars indicating the insertion region in intron 7. Different genomes show small variations in this insertion.

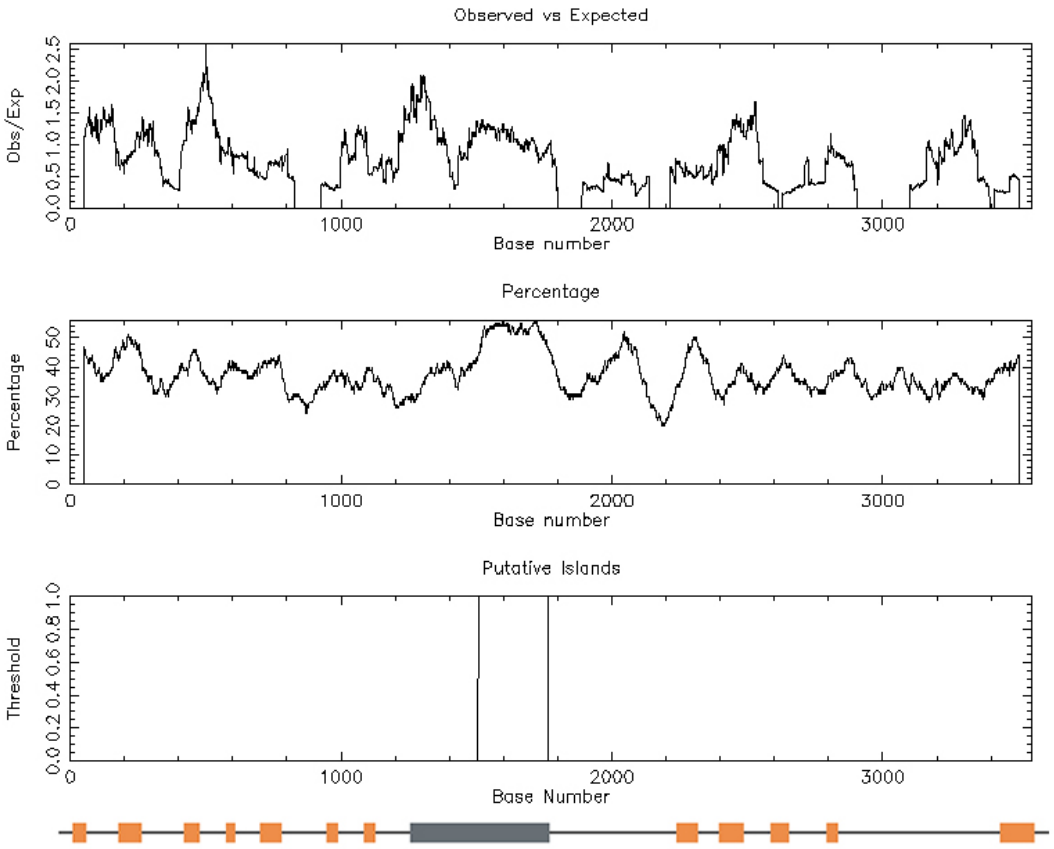

Fig. S7. Identification of CpG islands in the *Jc-JmMIEL1* H allele. Orange rectangles represent coding sequence and the gray rectangle indicating the *Copia* insertion region. Putative CpG islands were predicted in the *Copia* insertion region.

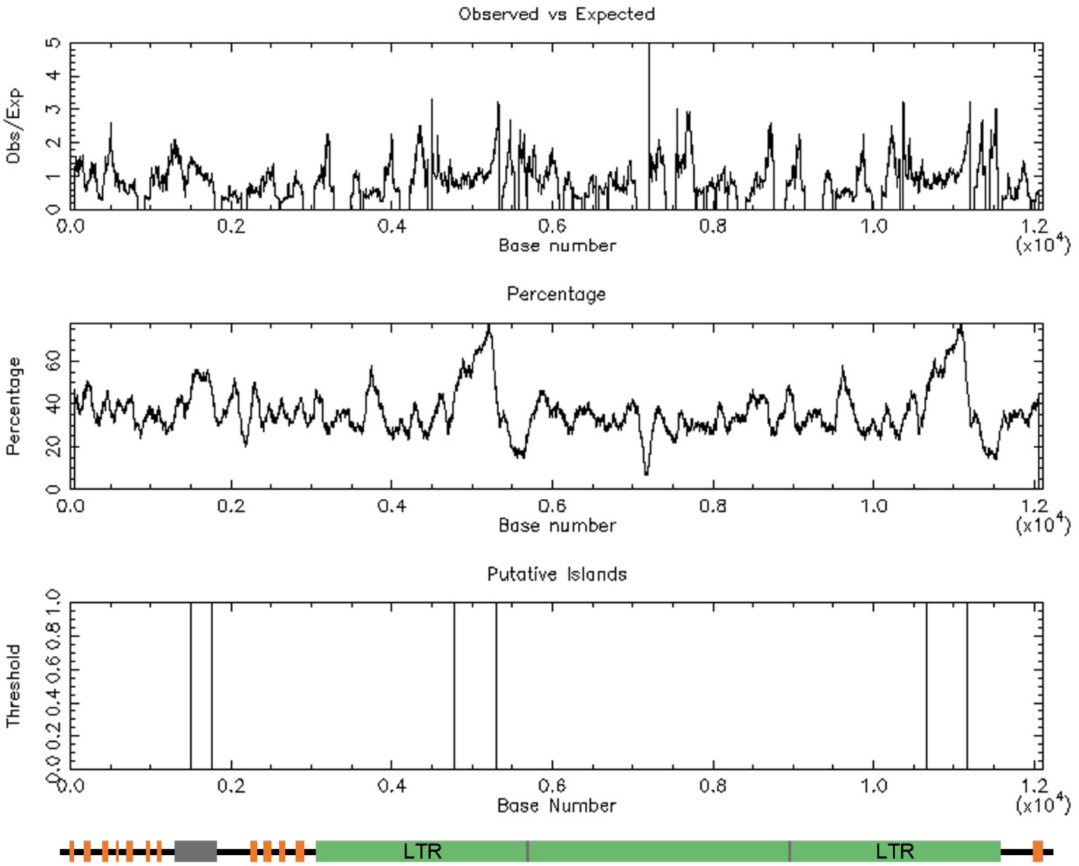

Fig. S8. Identification of CpG islands in the *Jc-JmMIEL1* L allele. The orange rectangle represents the coding sequence, the gray rectangle indicating the *Copia* insertion, and the green rectangle indicating the *Gypsy* insertion. Three putative CpG islands were identified within the *Copia* and *Gypsy* insertions, located in introns 7 and 11.

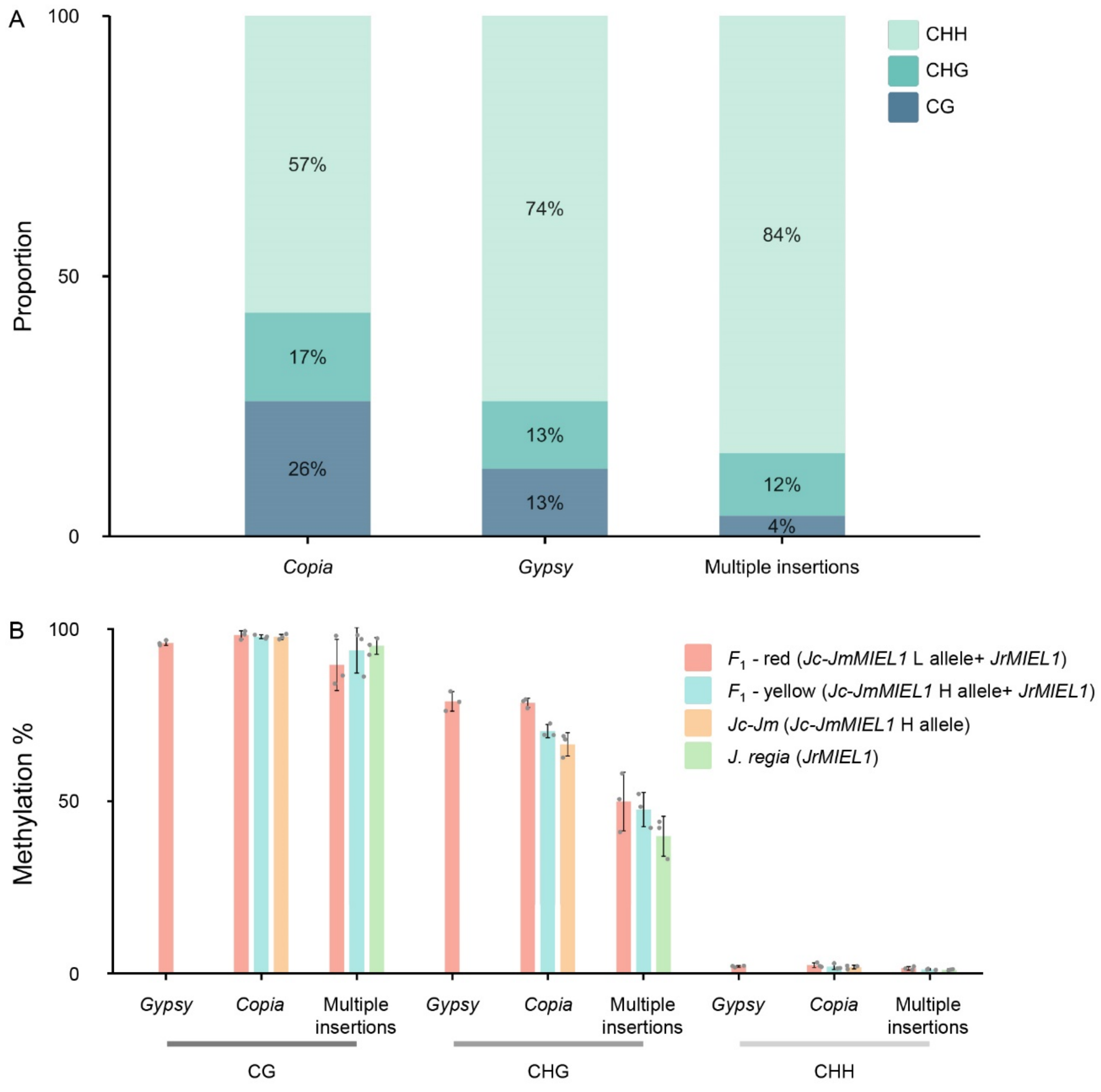

Fig. S9. Proportion of cytosine context and its methylation levels in different allele transposon insertions. (A) Proportion of cytosine context in different transposon insertion regions. (B) Cytosine methylation levels in different context of different transposon insertions in three *MIEL1* alleles. F_1 red includes *Jc-JmMIEL1* L and *JrMIEL1* allele, while F_1 yellow includes *Jc-JmMIEL1* H and *JrMIEL1* allele. *Jc-Jm* contains only the *Jc-JmMIEL1* H allele, and *J. regia* contains only the *JrMIEL1* allele. Additionally, the *Jc-JmMIEL1* L allele contains both *Copia* and *Gypsy* insertions, the *Jc-JmMIEL1* H allele contains only *Copia* insertions, and *JrMIEL1* contains multiple insertions.

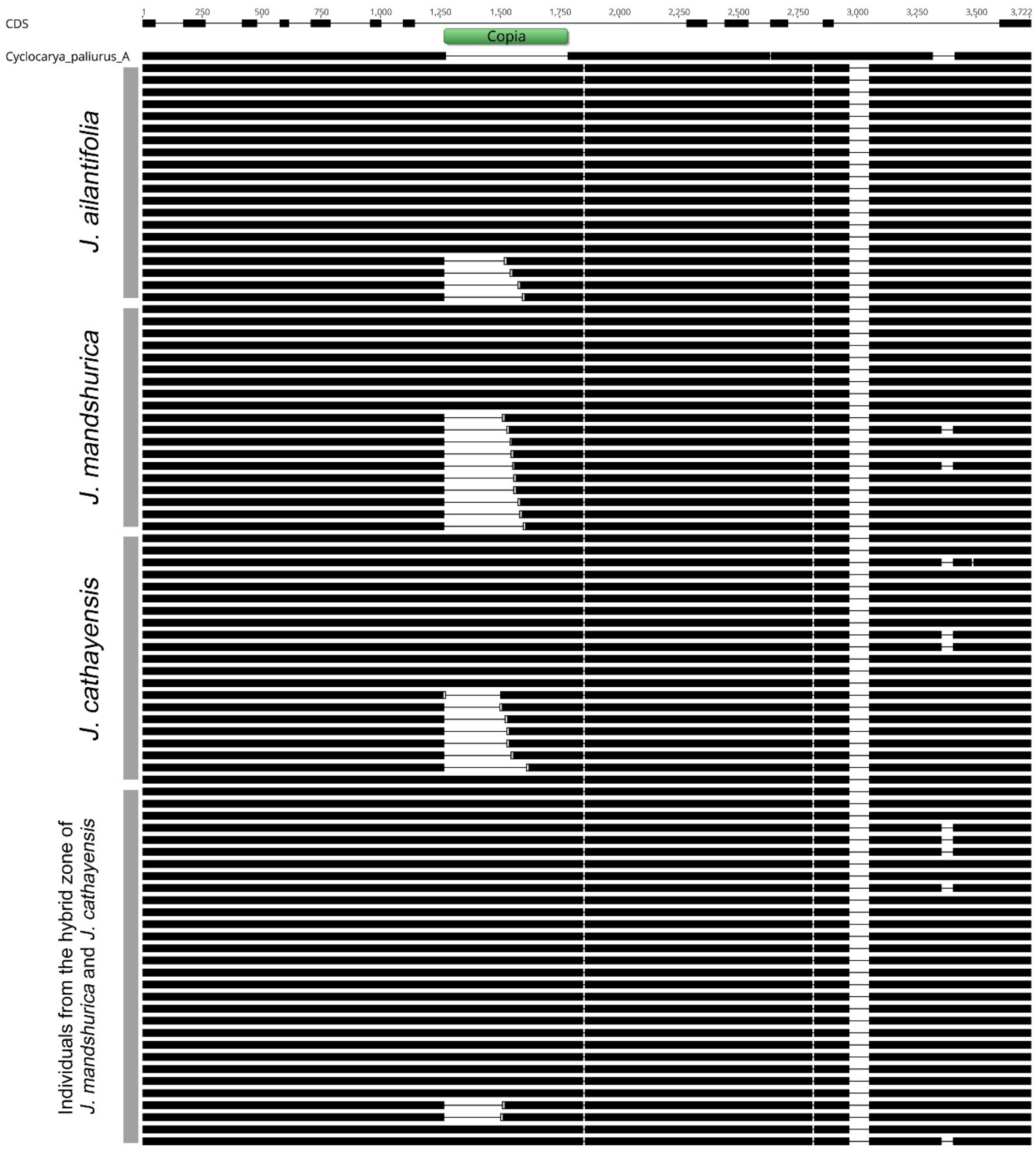

Fig. S10. *Copia* insertions identified in *J. ailantifolia*, *J. mandshurica*, *J. cathayensis*, and hybrid individuals from the hybrid zone of *J. mandshurica* and *J. cathayensis* (Xu et al. 2021) based on whole-genome resequencing data. The green rectangle represents the *Copia* insertion. Most individuals exhibited the 517 bp *Copia* insertion. However, due to the limitations of next-generation sequencing data, some individuals' *Copia* transposons were not fully assembled.

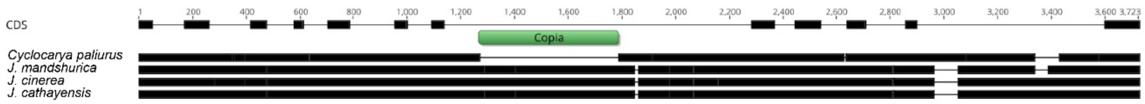

Fig. S11. The *Copia* insertion in *J. cinerea*, *J. cathayensis*, and *J. mandshurica* based on their reference genomes as cited in the main text and using *Cyclocarya paliurus* as the outgroup. The green rectangle represents the *Copia* insertion.

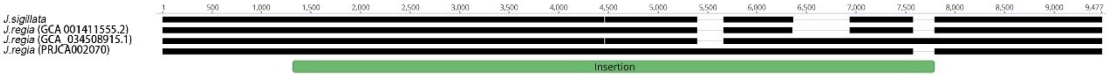

Fig. S12. Intron insertions in *J. regia* and *J. sigillata* based on their reference genomes as cited in the main text. Different genomes show small variations in this insertion.

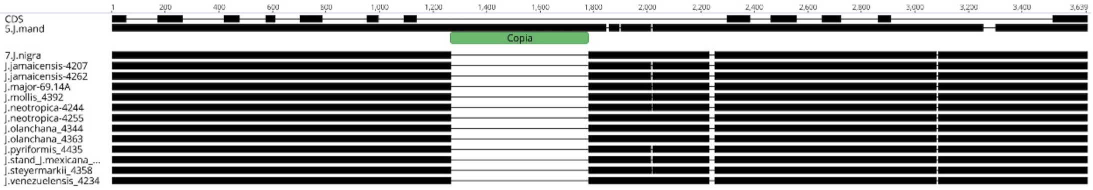

Fig. S13. The *Copia* insertion in *J. jamaicensis*, *J. major*, *J. mollis*, *J. neotropica*, *J. olanchana*, *J. pyriformis*, *J. mexicana*, *J. steyermarkii*, and *J. venezuelensis* (all with yellow stigmas) based on whole-genome sequencing data as cited in the main text. No *Copia* or *Gypsy* insertions were detected in any of these nine species.

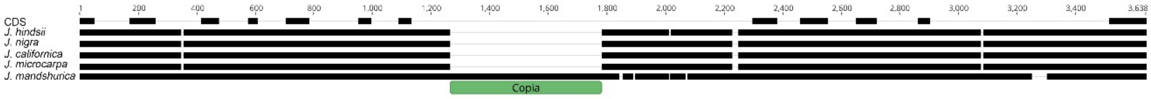

Fig. S14. Absence of a *Copia* insertion in *J. hindsii*, *J. nigra*, *J. californica* (with variable stigma color), and *J. microcarpa* (all with yellow stigmas), using *J. mandshurica* (with red stigmas) as the reference. No *Copia* insertions were detected in these four species.

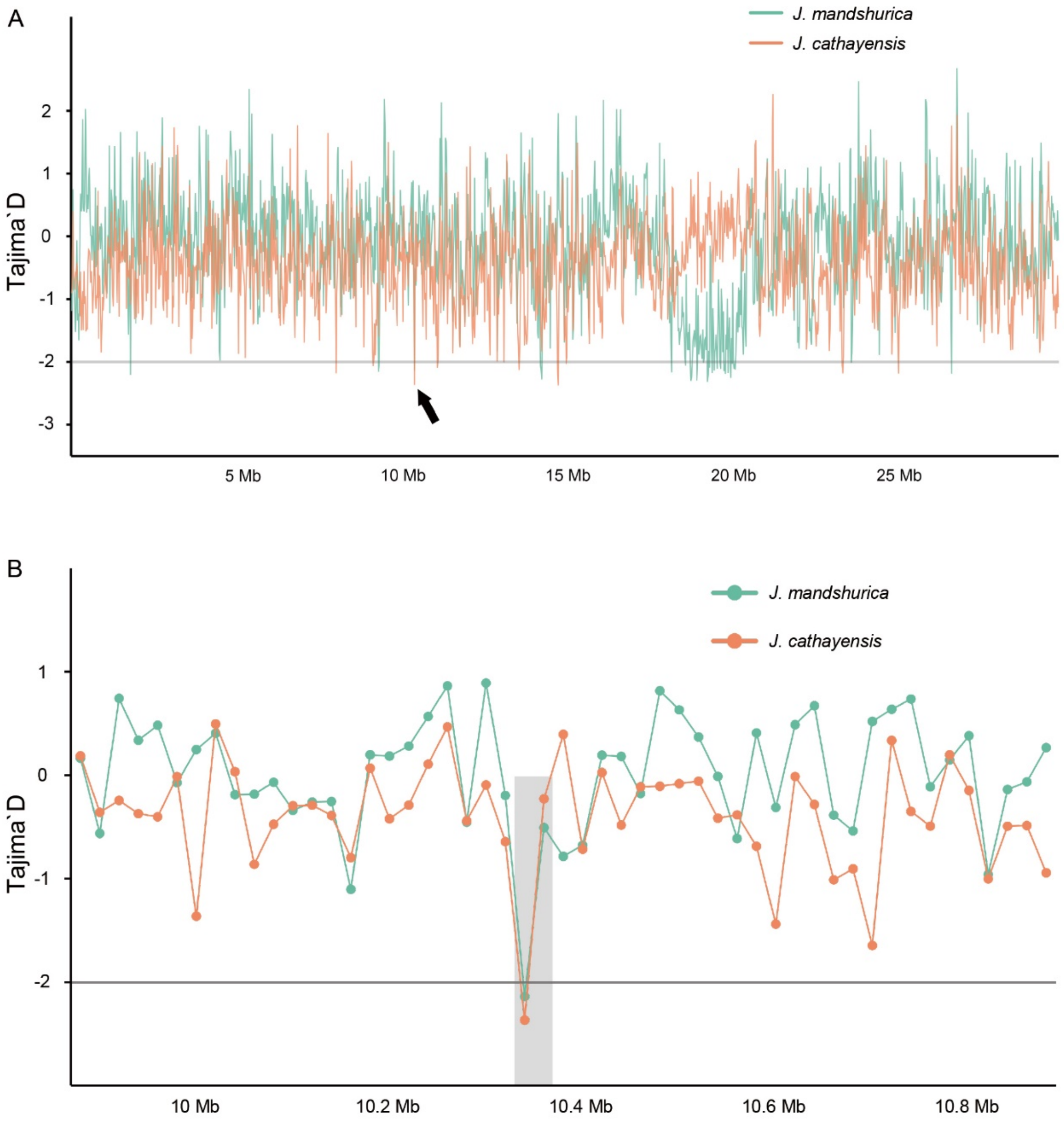

Fig. S15. Genome-wide Tajima`s D test results in 20-kb sliding windows across chromosome 10. (A) The overall distribution of Tajima's D values across chromosome 10, with the gene's position marked by arrows. (B) The Tajima's D distribution in the vicinity of the *MIEL1* gene, with its position highlighted in gray.

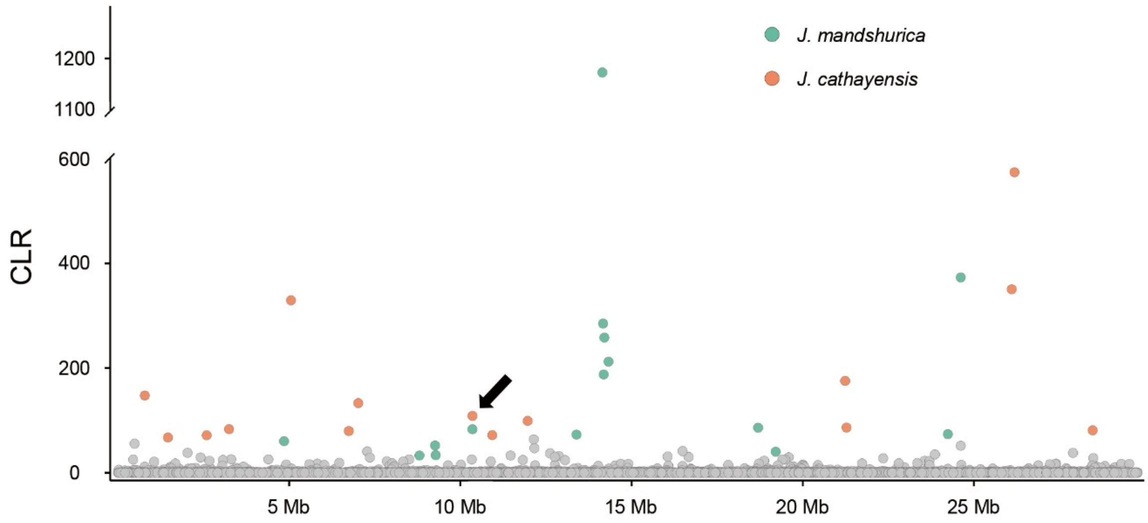

Fig. S16. Genome-wide *SweeD* result with 1500 grids positions across chromosome 10. Colored points represent the top 1% of grids with the highest CLR values. The position of the *MIEL1* gene is indicated by arrows.

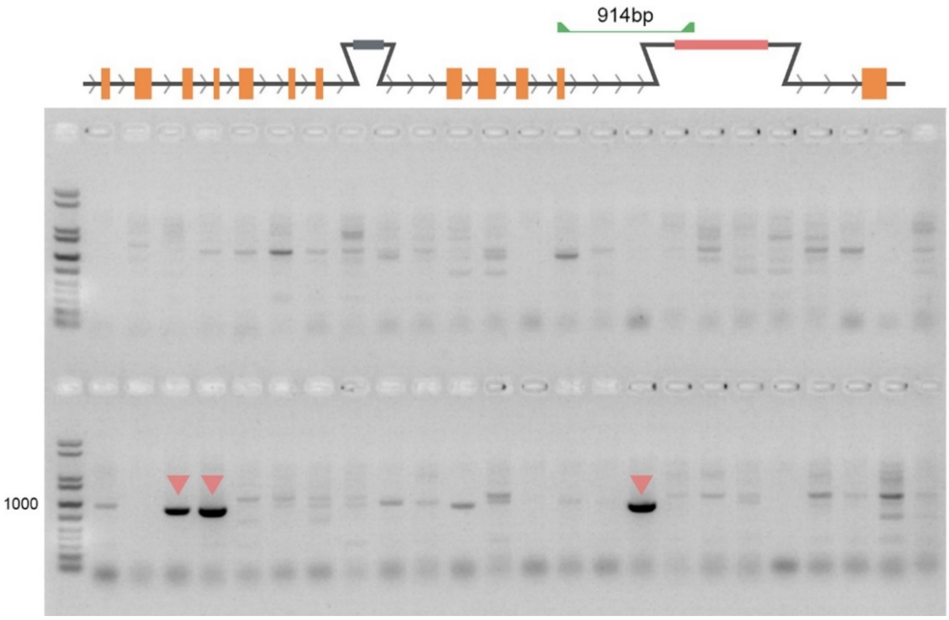

Fig. S17. *Gypsy* insertion confirmation with PCR amplification in hybrid individuals of the red-stigma species *J. cathayensis* and *J. mandshurica*. Primer locations are shown at the top, with orange indicating coding regions, pink indicating *Gypsy* insertions, and green indicating primer amplification regions. Three individuals with *Gypsy* insertions are indicated by pink arrows.

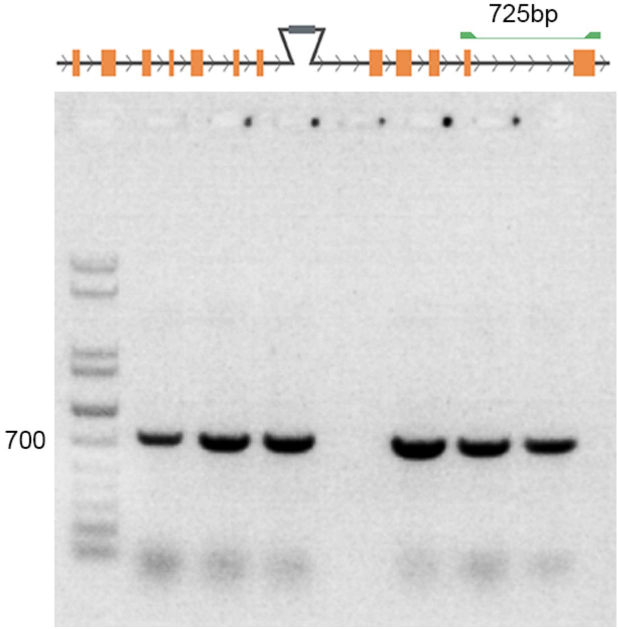

Fig. S18. Identification of heterozygous or homozygous *Gypsy* insertions by PCR amplification. Primer locations are shown at the top, with orange indicating coding regions and green indicating primer amplification regions. The left three lanes show the amplification results of heterozygous individuals with the *Gypsy* insertion in intron 11, while the right three lanes are the controls. Three individuals previously identified as having *Gypsy* insertions also had a 725 bp fragment of intron 11, indicating that their *Gypsy* insertions are heterozygous.

Table S1. Assignment of the RNA-sequencing reads obtained from F_1 hybrids to their parents.

Tissue	Stigma color	% unambiguous reads to *J. regia* genome	% unambiguous reads to *J. mandshurica* genome	Sample ID
catink	yellow	40.5028	40.1684	1
catink	yellow	40.7040	40.5324	2
catink	yellow	44.3012	37.7775	4
catink	yellow	41.1159	40.6860	6
catink	red	41.6156	40.3382	7
catink	red	43.8512	38.5378	8
catink	red	44.0531	38.3714	9
catink	red	41.5549	40.3674	11
leaf	yellow	39.9537	38.8436	1
leaf	yellow	40.1143	38.9636	2
leaf	yellow	39.8678	39.4261	4
leaf	yellow	40.1777	39.0383	6
leaf	red	40.0135	39.6489	7
leaf	red	40.1387	39.5303	8
leaf	red	40.1982	39.7091	9
leaf	red	39.8068	39.1352	11
ovary	yellow	40.1732	39.1016	1
ovary	yellow	40.2422	39.0010	2
ovary	yellow	41.5311	38.9693	4
ovary	yellow	40.1786	39.6931	6
ovary	red	40.1606	39.3880	7
ovary	red	40.0960	39.5477	8
ovary	red	39.8346	39.4147	9
ovary	red	39.9261	39.3988	11
stigma	yellow	39.8340	39.4161	1
stigma	yellow	39.8031	39.3311	2
stigma	yellow	39.8191	40.5108	4
stigma	yellow	40.1071	40.4496	6
stigma	red	39.8256	39.9030	7
stigma	red	39.5290	41.0451	8
stigma	red	39.9273	40.1242	9
stigma	red	39.5734	39.9544	11

Table S2. Correlation coefficients between *MIEL1* and homologs of the anthocyanin-structural genes in *Juglans*: Anthocyanidin Reductase (ANR), Chalcone-Flavanone Isomerase (CHI), and Flavonone 3-Hydroxylase (F3H).

Gene	SeqID	Spearman's correlation with MIEL1/FDR	Pearson's correlation with MIEL1/FDR
JrANR	JreChr09G12363	-0.6556/1.02E-03	-0.6943/1.67E-04
JrCHI	JreChr01G13213	-0.6113/1.87E-03	-0.7233/1.57E-04
JrF3H	JreChr07G12902	-0.6721/1.02E-03	-0.7090/1.57E-04
*Jc-Jm*ANR	Jman008T0188100	-0.7613/1.65E-05	-0.6679/3.08E-04
*Jc-Jm*CHI	Jman001T0028000	-0.5112/4.52E-03	-0.6321/3.08E-04
*Jc-Jm*F3H	Jman005T0068900	-0.6376/3.84E-04	-0.6358/3.08E-04

Table S3. Differentially expressed genes in the anthocyanin pathway across *J. regia* and the hybrid individuals of *J. mandshurica* and *J. cathayensis*.

Anthocyanin 5-O-Glucosyltransferase (A5GT), anthocyanin 5-glucoside malonyl transferase (5MAT), Flavonone 3-Hydroxylase (F3H), Flavonoid 3'-Hydroxylase (F3'H), Chalcone-Flavanone Isomerase (CHI), Anthocyanidin Synthase (ANS), and Anthocyanidin Reductase (ANR)

Gene	SeqID	logFC	logCPM	LR	PValue	FDR
A5GT	Jman001T0230200.1	6.4084	5.1772	79.4332	4.99E-19	2.84E-17
5MAT	Jman006T0058400.1	1.3197	6.5604	10.7109	1.07E-03	5.16E-03
F3H	Jman005T0068900.1	0.9790	9.8479	8.2609	4.05E-03	1.61E-02
F3'H	Jman004T0063500.1	0.9176	8.3563	7.1033	7.69E-03	2.78E-02
CHI	Jman001T0028000.1	2.6181	8.9778	77.1157	1.61E-18	8.75E-17
ANS	Jman004T0201800.1	2.2311	8.9585	23.7199	1.11E-06	1.19E-05
ANR	Jman008T0188100.1	0.9709	8.0862	4.01988	0.04496	0.1193

Table S4. Primer sequences used in this study.

Primer name	sequence
Jc_JmMIEL1_act97_F	CTGGCCGTGATCTTACTGATG
Jc_JmMIEL1_act97_R	TCTTTGCGGTCTCAAGTTCCTG
Jc_JmMIEL1_MIEL1_F	GCGTGACAAGTATTGCTGTCC
Jc_JmMIEL1_MIEL1_R	TCGGTAATCCTCCGGCATGA
J. nigra_act97_F	CTGGCCGTGATCTTACCGAT
J. nigra_act97_R	TCTTTGCGGTCTCAAGTTCCTG
J. nigra_MIEL1_F	GCGTGACAAGTATTGCTGTCC
J. nigra_MIEL1_R	TCGGTAATCCTCTGGCATGAC
J. regia_act97_F	CTGGCCGTGATCTTACCGAT
J. regia_act97_R	GCTCTTTGCGGTCTCAAGTTCT
J. regia_MIEL1_F	GCGTGACAAGTATTGCTGTCC
J. regia_MIEL1_R	TCTGTAATCCTCCGGCATGAC
Jc-Jm-eYFP-F	CATTTGGAGAGGACACGCTCGAGATGGAAGGCTCAT CCAACGAAC
Jc-Jm-eYFP-R	TCCTCGCCCTTGCCCATGGATCCCTGAGGAAGAACT GGAGGTGC
Copia-F	TCTTATGTTAAATCGAACTTACCATTTCCTGTTTTGTT TTGCAAATCC
Copia-R	AAAACAAAACAGGAAATGGTAAGTTCGATTTAACATA AGATCATTAGAA ATTAACAGAC
Intron7-F	TGCTCTTTCCCTAATACTAAACAGAAG
GPS-CDS11-R	CCACATGATACTGCCTGGTTTG
MIEL1-End-R	TCCTGTAATTGTAGTCTGATCACTGA
Gypsy-LTR1-R	GATTGGATCTCATTTCTTTCCCTAC
CDS11-F	CGGAGGATTACCGATACAGGA
LTR-1-F	GTGTTTGGCTGTAACTGCACA
LTR-1-R	GTTGAGCTACCTCGTACTCAATG
LTR-2-F	CCATAATCATCTAGCTTGTAGTCCT
